# Analysis of sensory system aspects of postural stability during quiet standing in adolescent idiopathic scoliosis patients

**DOI:** 10.1186/s12984-018-0395-6

**Published:** 2018-06-22

**Authors:** Taeyong Sim, Hakje Yoo, Dongjun Lee, Seung-Woo Suh, Jae Hyuk Yang, Hyunggun Kim, Joung Hwan Mun

**Affiliations:** 1 Department of Bio-Mechatronic Engineering, College of Biotechnology and Bioengineering, Sungkyunkwan University, Natural Sciences Campus, 2066 Seobu-ro, Jangan-gu, Suwon-si, Gyeonggi-do 16419 South Korea; 2 Department of Orthopedics, Scoliosis Research Institute, Korea University Medical College, Guro Hospital, 148 Gurodong-ro, Guro-gu, Seoul, 08308 South Korea

**Keywords:** Adolescent idiopathic scoliosis (AIS), Discrete wavelet transform (DWT), Postural stability, Balance, Sensory system, Visual, Vestibular, Somatosensory

## Abstract

**Background:**

The aim of this study was to quantitatively analyze quite standing postural stability of adolescent idiopathic scoliosis (AIS) patients in respect to three sensory systems (visual, vestibular, and somatosensory).

**Method:**

In this study, we analyzed the anterior-posterior center of pressure (CoP) signal using discrete wavelet transform (DWT) between AIS patients (*n* = 32) and normal controls (*n* = 25) during quiet standing.

**Result:**

The energy rate (*∆E*_*EYE*_%) of the CoP signal was significantly higher in the AIS group than that in the control group at levels corresponding to vestibular and somatosensory systems (*p* < 0.01).

**Conclusions:**

This implies that AIS patients use strategies to compensate for possible head position changes and spinal asymmetry caused by morphological deformations of the spine through vestibular and somatosensory systems. This could be interpreted that such compensation could help them maintain postural stability during quiet standing. The interpretation of CoP signal during quiet standing in AIS patients will improve our understanding of changes in physical exercise ability due to morphological deformity of the spine. This result is useful for evaluating postural stability before and after treatments (spinal fusion, bracing, rehabilitation, and so on).

## Background

Adolescent idiopathic scoliosis (AIS) is defined as a three-dimensional morphological deformity of the spine [1]. The severity of AIS is generally diagnosed by measuring Cobb’s angle, the golden standard with 10 degrees diagnostic criteria on radiograph based X-ray images [1, 2]. The morphological deformity of the spine is intimately interconnected with the central-nervous system [3, 4], altering the sensory process [5–7] and causes balance dysfunction of the static stability [8–10]. The morphological deformity such as concave (the inside of scoliosis curve direction) and convex (the outside of scoliosis curve direction) of the spine can cause alternation in head position [11, 12] and asymmetric activity of the muscles surrounding the spine [13], which can result in trunk center of mass (CoM) change [14]. People with spinal deformity maintain their postural stability by rearranging these alterations through various interactions of sensory systems [11, 15, 16]. The processing for controlling postural instability is determined by sensory organization of visual, vestibular, and somatosensory systems [17]. Therefore, sensory processing of postural stability during quiet standing in AIS patients can be an important analytical factor [18–20].

The human body is designed to maintain a vertical orientation to the eyes and ground parallel in horizontal plane [21]. To maintain postural stability, the visual system adjusts the posture by providing information about the surrounding environment [17] while vestibular system adjusts the posture by managing the movement and position of the head [22]. In other words, both visual and vestibular systems help adjust the spatial orientation for maintaining postural stability [23, 24]. The somatosensory system helps support the body to maintain postural stability; additionally, provides information on the improvement of joint stability to maintain the static state through controlling muscle activation by the nervous system [25–27]. This kinematic control ability is organized in close association with the input from the visual and vestibular systems [23, 28]. The analysis of the center of pressure (CoP) can be applied to interpret a postural instability with respect to sensory processing during quiet standing in AIS patients [18, 29, 30]. The CoP signal is distinguished by visual (< 0.1 Hz), vestibular (0.1–0.5 Hz), and somatosensory (0.5–1.0 Hz) systems according to the frequency [31–34]. In fact, different power spectrum intervals have been reported in various studies, including 0–0.5, 0.5–2, and > 2 Hz [35]; 0–0.1, 0.1–0.5, and 0.5–1 Hz [31], and 0–0.3, 0.3–1, and 1–3 Hz [36]. However, according to recent studies, criteria of 0–0.1, 0.1–0.5, and 0.5–1 Hz are frequently used to analyze the effect a certain medicine treatment on the postural stability of patients with unilateral vestibular disorder [34], evaluate postural sway dynamics in quiet standing [32], and investigate balance functions in migraineur with and without vertigo [33]. These criteria correspond to each sensory system were calculated by measuring oscillations made on a stabilometer in a second [31].

A sensory organization test (SOT) has been used to evaluate sensory information while maintaining postural stability in AIS patients [8–10]. The SOT method is used to observe posture control during standing according to environmental changes of the three sensory systems (visual, vestibular, and somatosensory) [8–10]. The SOT method evaluates the postural control ability in six states. Individual sensory information is identified through comparative analysis of two of these six states. For example, vestibular sensory information is evaluated by dividing the result of the protocol that is responsible for the vestibular system into results of the protocol of the three sensory systems [9]. The SOT has been used to analyze the effect of a spinal brace on postural control for AIS [8], the difference in sensory information according to the degree of spinal deformation [9], and the relationship between somatosensory function changes and postural control in AIS patients [10]. However, using SOT to quantify the functions of visual, vestibular, and somatosensory systems during quiet standing has some limitations. First, a spatial constraint arises due to the size of the equipment used to analyze the sensory system, for example, Smart Equitest (NeuroCom International Inc., Clackamas OR, USA): size = 1350 × 1550 × 2390 mm. Second, to identify the complex sensory integration, sensory information should be acquired simultaneously. Consequently, the individual results can be obtained from two separate SOT experiments [8–10]. This method also has limitations in multiple analysis of the sensory integration process for maintaining the stability of standing posture [8–10].

In this study, we applied discrete wavelet transform (DWT) method to analyze the sensory integration process, using only CoP signal without additional equipment, in order to evaluate postural instability during quiet standing. DWT method is used to decompose a signal composed of high and low frequencies. It is appropriate to interpret effects of three sensory systems (visual, vestibular and somatosensory systems) on maintaining postural stability through decomposing the CoP signal [37]. Additionally, we evaluated sensory information characteristic in postural instability depending on AIS severity according to Cobb’s angle.

## Methods

### Participants

Fifty-seven subjects participated in this study to analyze the effect of morphologic deformation on postural instability, including 32 AIS patients (all females, age = 14.3 ± 2.2 yr., height = 155.8 ± 9.2 cm, weight = 44.0 ± 8.3 kg, cobb angle = 31.1 ± 15.8°) without history of surgical treatment or rehabilitation and 25 adolescents (all females, age = 14.8 ± 4.2 yr., height = 156.8 ± 6.3 cm, weight = 44.2 ± 7.6 kg) without musculoskeletal diseases of similar age. In order to analyze posture control according to AIS severity, we divided the subjects into three cases of severity based on the largest Cobb’s angle (10° < mild ≤20° (*n* = 12); 20° < moderate ≤40° (*n* = 10); and severe > 40° (n = 10) [38, 39]. All experimental protocols were approved by the Institutional Review Boards of Korea University Guro Hospital. All experiments were performed in accordance with relevant approved guidelines and regulations of Sungkyunkwan University. Before the experiment, all participants provided informed consent to participate.

### Experimental setup and data collection

We used anterior-posterior CoP during quiet standing to analyze the effect of spinal morphologic deformity on postural instability. The CoP defined as the point location of the ground reaction force vector has been used as a parameter for evaluating balance control ability of CoM [40]. Subjects stood upright in a comfortable stance, and they were asked to hold their arms along the side of their body while standing quietly on a force plate. The standing condition was divided into two conditions during quiet standing (with eyes open and with eyes closed) in order to analyze the difference in the sensory system according to change of visual information [37]. Differing eyes open and eyes closed conditions, the presence and absence of visual information, an important design parameter, allows observers to measure activities of vestibular and somatosensory systems in postural control [37, 41]. A CoP signal in eyes open condition was used to analyze the effect of normal sensory information from visual, vestibular, and somatosensory systems on posture control. On the contrary, that from eyes closed condition facilitated the analysis of the effect of vestibular and somatosensory information on posture control with limited visual information.

In addition, difference of energy contents between eyes open and eyes closed conditions could allow quantitative analysis about the effect of differences among three sensory systems on posture control. Subjects were asked to stare at a dot marked at a horizontal distance from their eyes. In the eyes open experiment, subjects kept staring at the dot. In the eyes closed experiment, subjects instantly closed their eyes at the start of the experiment [37]. Each subject continued quiet standing with eyes open or with eyes closed for a period of 70s [42]. This trial was repeated six times. Average values of each eyes open and eyes closed condition were used. CoP data were obtained using force plates (OR6–2000, AMTI Inc., Newton, Massachusetts, USA) and sampled at 120 Hz [43].

### Data processing

To compare the difference in sensory system between AIS and control groups, CoP signal was analyzed using a DWT to compare between conditions of eyes open and eyes closed during quiet standing [37]. DWT can be decomposed into a high frequency and a low frequency signal through the scaling function (φ_(*j*, *k*)_(*t*)) and the wavelet function (*ψ*_(*j*, *k*)_(*t*)), respectively (Eq. (1)).

1 $$ f(t)=\sum \limits_k\sum \limits_j^{\infty }{c}_{j,k}{\upvarphi}_{j,k}\left(\mathrm{t}\right)+\sum \limits_k\sum \limits_j^{\infty }{d}_{j,k}{\psi}_{j,\mathrm{k}}\left(\mathrm{t}\right) $$

In the above Equation, *c*_*j*, *k*_ and *d*_*j*, *k*_ denote approximated coefficients and detailed coefficients, respectively. *j* and *k* denote decomposition level (*j* = 1, 2, …, *J*) and discrete location of the CoP signal (*f*(*t*))*.* The detail signal (*W*(*j*, *k*)) and approximated signal (*S*(*j*, *k*)) of the CoP signal are obtained using the following Eqs. (2) and (3).

2 $$ W\left(j,k\right)={\int}_{-\infty}^{\infty }f(t){\psi}_{j,k}\left(\mathrm{t}\right) dt $$

3 $$ S\left(j,k\right)={\int}_{-\infty}^{\infty }f(t){\upvarphi}_{\left(j,k\right)}(t) dt $$

From the Eqs. (4) and (5), energy content (*E*(*j*)) and total energy content (*E*_*T*_) of the corresponding decomposition level are calculated using detail signal and approximated signal obtained above, respectively. Where, *K* (*j*) is discrete location in level *j* [37].

4 $$ E(j)=\sum \limits_{k=0}^{K\ (j)}{\left(W\left(j,k\right)\right)}^2 $$

5 $$ {E}_T=\sum \limits_{k=0}^{K\ (j)}{\left(S\left(j,k\right)\right)}^2+\sum \limits_{j=1}^J\sum \limits_{k=0}^{K\ (j)}{\left(W\left(j,k\right)\right)}^2 $$

The energy content in eyes closed case (*E*_*EC*_ % (*j*)) is calculated as a percentage of the ratio between total energy content (*E*_*T*_) and energy content corresponding to decomposition levels (*E*_*EC*_(*j*)). The energy content in eyes open case (*E*_*EO*_ % (*j*)) is calculated using the same method (Eqs. (6) and (7)).

6 $$ {E}_{EC}\%(j)=\left(\frac{\left({E}_{EC}(j)\right)}{E_T}\right)\ast 100\% $$

7 $$ {E}_{EO}\%(j)=\left(\frac{\left({E}_{EO}(j)\right)}{E_T}\right)\ast 100\% $$

In order to analyze energy content differences between eyes closed and eyes open conditions, energy rate (*∆E*_*EYE*_(*j*)) was quantified by the following Eq. (8).

8 $$ \Delta  {E}_{EYE}(j)=\left(\frac{E_{EC}\%(j)-{E}_{EO}\%(j)}{E_{EO}\%(j)}\right)\ast 100 $$

**Fig. 1:**
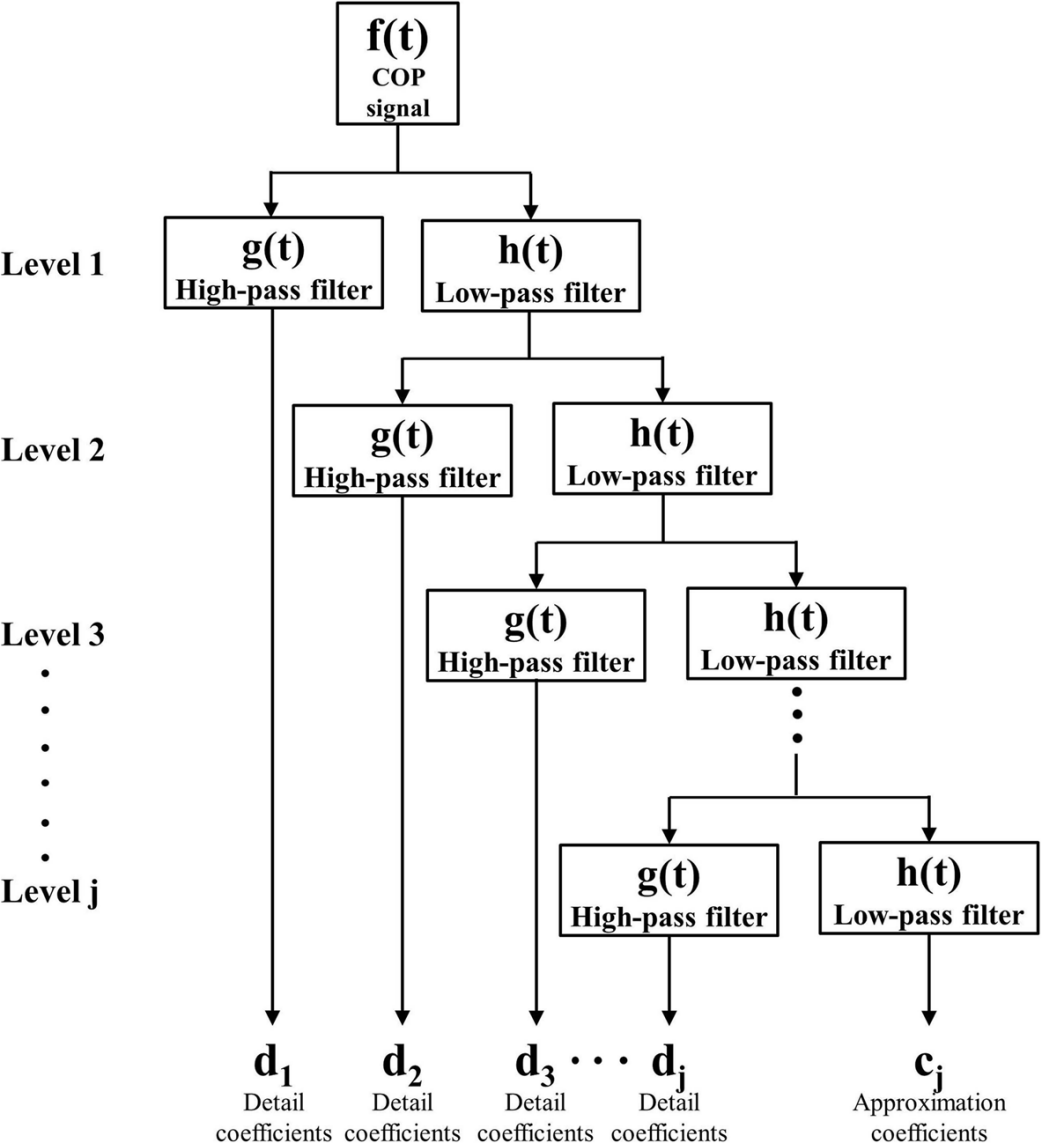
Diagram of analysis process of CoP signal using DWT method. The level of DWT is determined by the characteristic of CoP signal and wavelet function

Figure 1 presents the decomposition process of CoP signal using DWT. Decomposition level *j* is calculated considering the characteristics of both CoP signal and wavelet function. For each level, the CoP signal is decomposed by applying a high pass filter and a low pass filter. Detail coefficients (*d*_*1*_*~d*_*j*_) are obtained through level *1* to *j* high-pass filter while the approximation coefficient (*c*_*j*_) at level *j* is obtained through the low-pass filter.

### Statistical analysis

Statistical analysis was performed using SPSS 15.0 software (SPSS Inc., Chicago, IL, USA) for analyzing changes in the sensory system between conditions of eyes open and eyes closed during quiet standing in AIS and control groups. Energy contents using DWT were analyzed by calculating the average and standard deviations. To analyze the difference in energy contents between eyes open and eyes closed conditions in the same group and the difference in the energy rate between AIS and control groups, independent *t*-tests were performed for all variables. Analysis of variance (ANOVA) was performed on variables to analyze difference in energy rate between AIS patients with three different severity levels based on Cobb’s angle. Differences among the three groups of AIS were analyzed using Bonferroni post-*hoc* test. Statistical significance level was set at *p* value of less than 0.05.

## Results

### Discrete decomposition of CoP signal using DWT and analysis of sensory system in both control and AIS groups

**Fig. 2:**
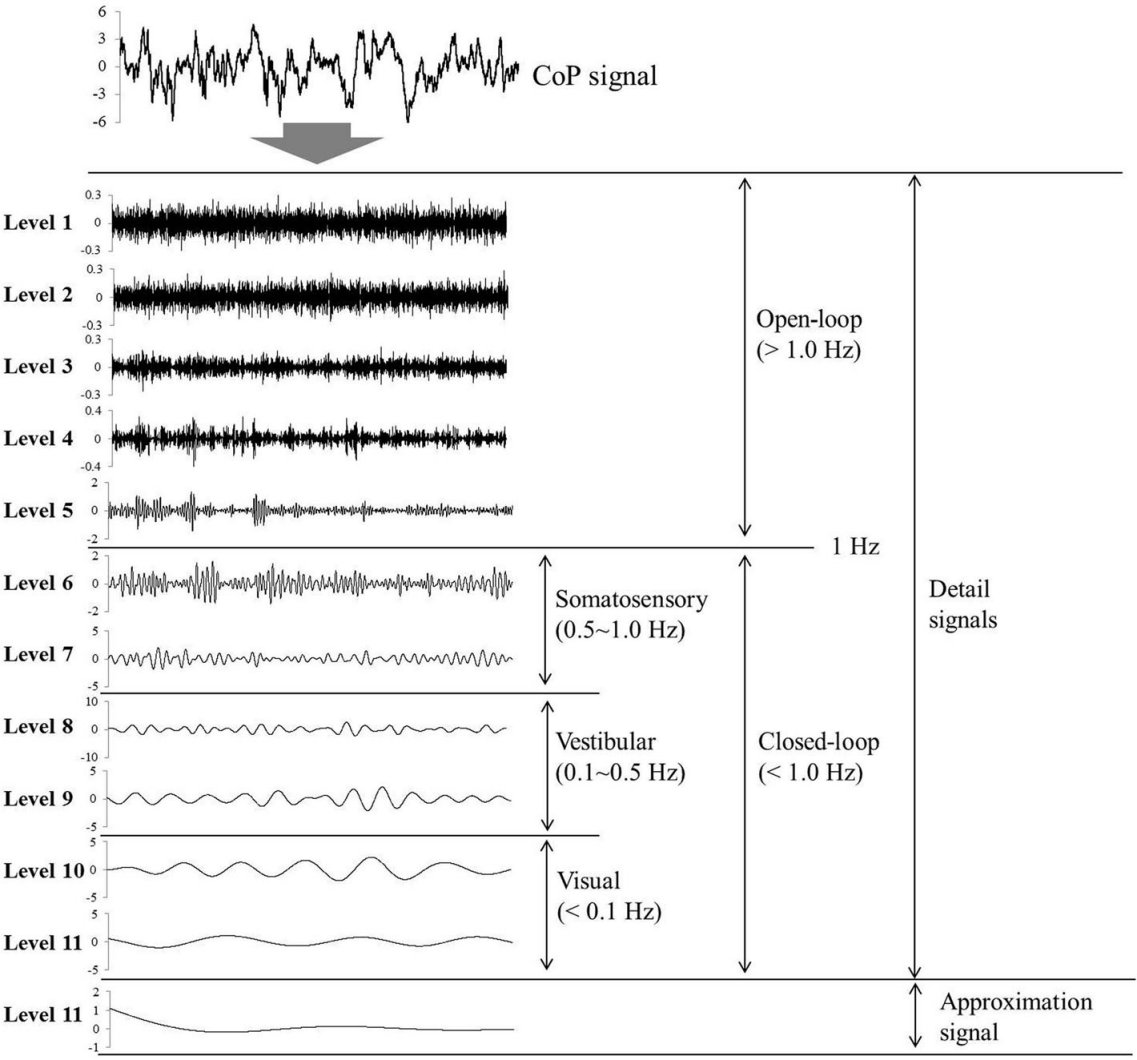
Results of the discrete decomposition of the CoP signal into 11 level low-frequency signals. This decomposition is the result of using 11 level discrete wavelet transform during quiet standing with eyes open in people without AIS. Based on level 5, open-loop and closed-loop were divided. The closed-loop (levels 6~ 10) group is divided into three sections (levels 6, 7: somatosensory (0.5~ 1.0 Hz); levels 8, 9: vestibular (0.1~ 0.5 Hz); levels 10, 11: visual (below 0.1 Hz))

To quantitatively analyze the sensory system known to control postural stability in AIS and control groups during quiet standing, we acquired ten detail coefficients for low frequency components and one approximate coefficient for high frequency components of the CoP signal using DWT. Figure 2 shows an example of reconstructing the results into low frequency signal and high frequency signal of CoP. The downward direction denotes an increasing discrete level. The signal is divided into an approximation signal (high frequency signal) and detail signal (low frequency signal). The open-loop and closed-loop signals are separated by 1 Hz [44]. In addition, the closed-loop signal is distinguished by three sensory systems (Somatosensory: 0.5–1.0 Hz; Vestibular: 0.1–0.5 Hz; Visual: < 0.1 Hz) according to the frequency range [31–34].

**Fig. 3:**
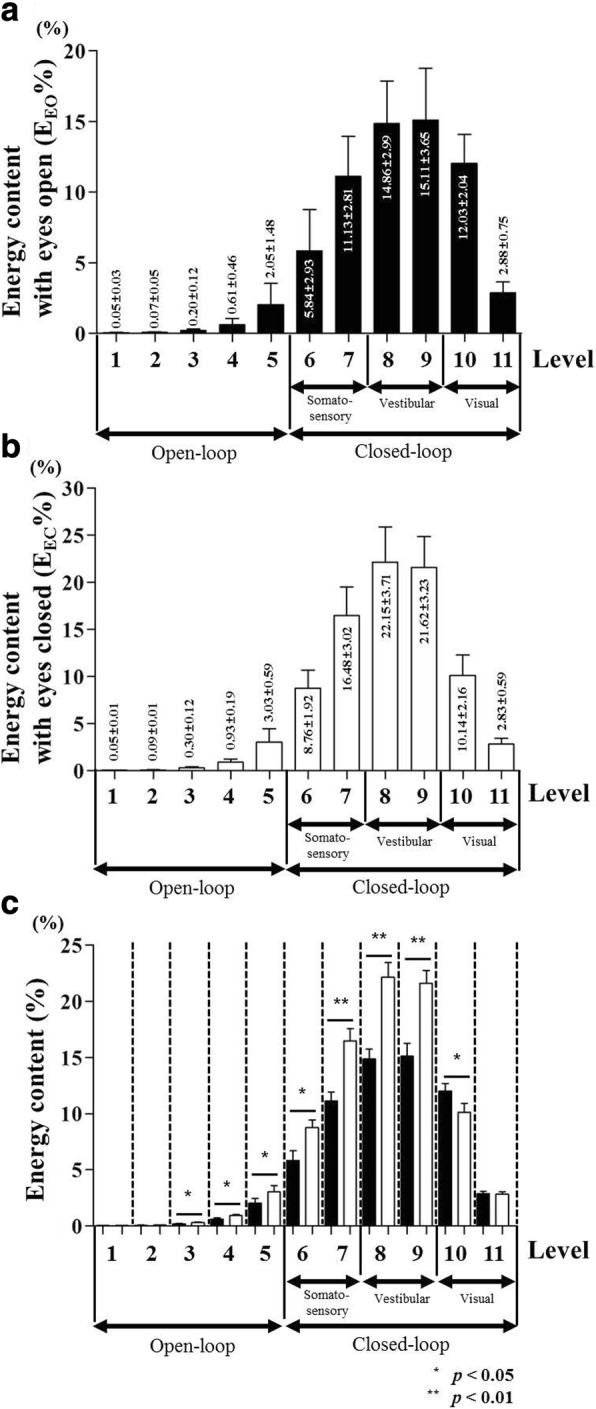
Energy content (%) of CoP signal during quiet standing in control group. **a** Energy content (*E*_*EO*_%) for each level section with eyes open condition (**b**) energy content (*E*_*EC*_%) for each level section with eyes closed condition (**c**) statistical comparison of difference between *E*_*EO*_% and *E*_*EC*_% for each level section

Figures 3a and b show results of transforming low frequency signals of CoP signals into energy content (%) during quiet standing with eyes open and eyes closed in the control group. The vertical axis shows the magnitude of energy content (%) in the corresponding frequency range. The magnitude of energy content (%) for each level section shows a similar value in eyes open and eyes closed subjects. The major part of the energy content (%) was represented in the closed-loop signal. The highest magnitude of energy content (%) in the closed-loop signal was shown at the vestibular section (eyes open = 29.97 ± 4.72%; eyes closed = 43.77 ± 4.92%), followed by that at the somatosensory section (eyes open = 16.97 ± 4.06%; eyes closed = 25.25 ± 3.58%) and the visual section (eyes open = 14.91 ± 2.18%; eyes closed = 12.97 ± 2.24%). Figure 3c shows difference in energy content (%) between eyes open and eyes closed subjects for each level section.

**Fig. 4:**
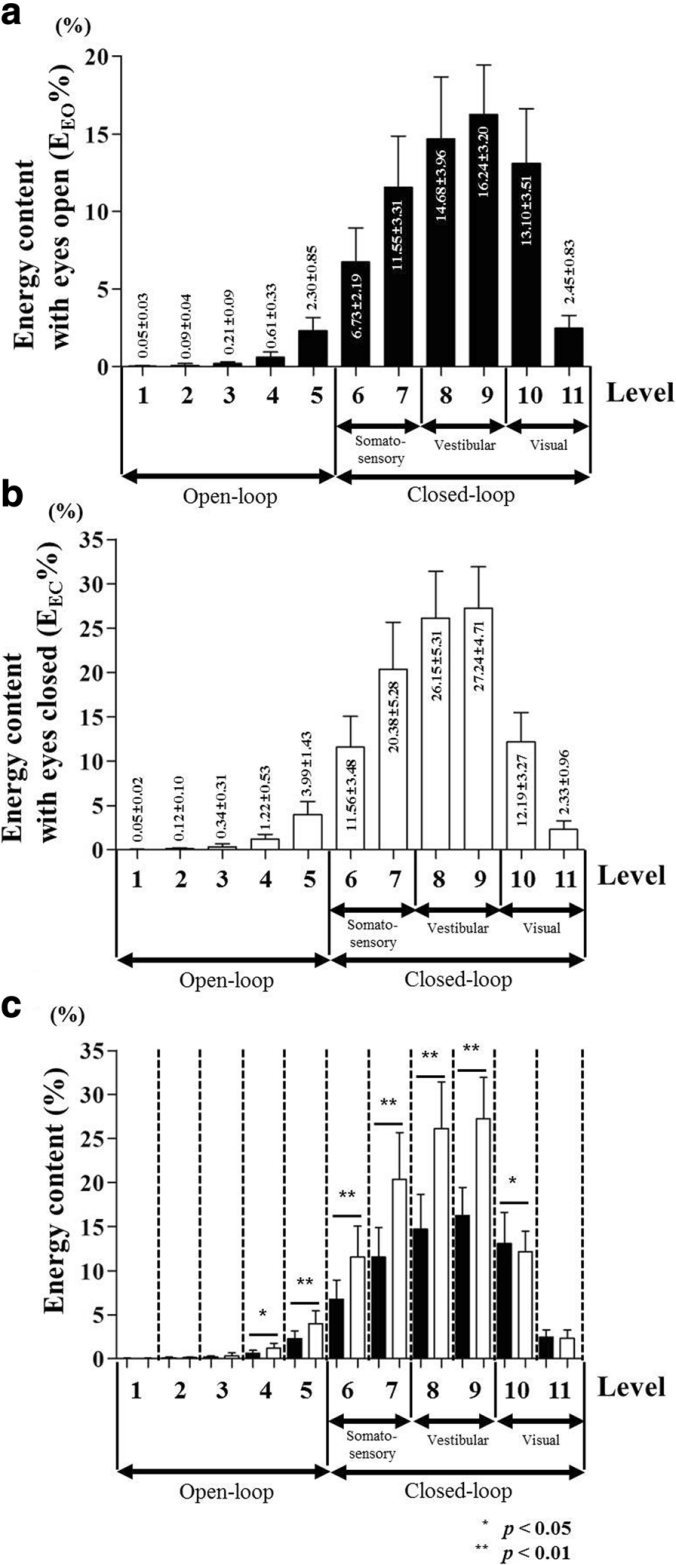
Energy content (%) of CoP signal during quiet standing in AIS group. **a** Energy content (*E*_*EO*_%) for each level section with eyes open condition (**b**) energy content (*E*_*EC*_%) for each level section with eyes closed condition (**c**) statistical comparison of difference of *E*_*EO*_% and *E*_*EC*_% for each level section

Figures 4a and b show results in the AIS group. The highest magnitude of energy content (%) was found at the vestibular section (eyes open = 30.93 ± 5.09%; eyes closed = 53.39 ± 7.09%), followed by that at somatosensory section (eyes open = 18.29 ± 3.97%; eyes closed = 31.94 ± 6.33%) and the visual section (eyes open = 15.55 ± 3.61%; eyes closed = 14.52 ± 3.41%). Figure 4c shows difference of energy content (%) between eyes open and eyes closed subjects for each level section. *E*_*EC*_% represents a significantly higher value than *E*_*EO*_% at levels 4~ 9 while *E*_*EO*_% shows a higher value than *E*_*EC*_% at level 10 (*p* < 0.05 or *p* < 0.01).

### Difference of energy content (%) between control and AIS in eyes open and eyes closed subjects

**Fig. 5:**
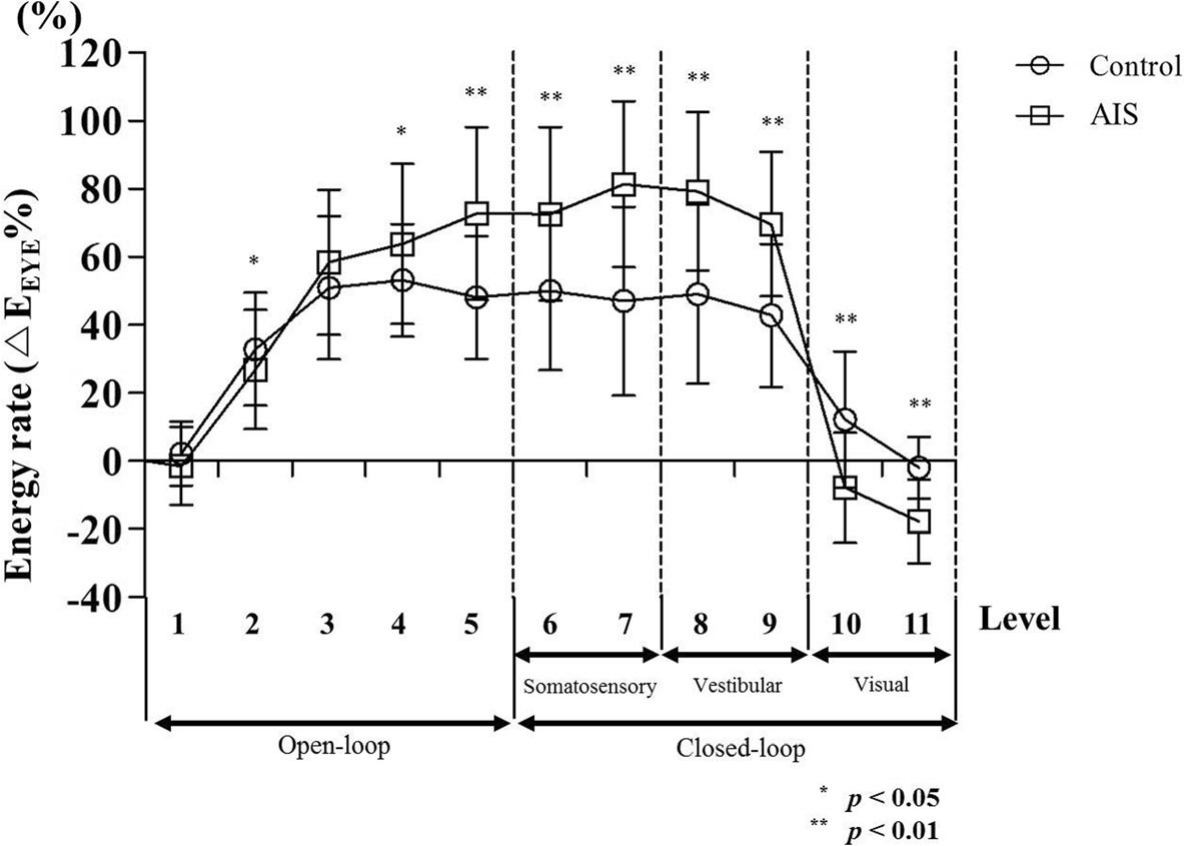
Difference in energy rate (*∆E*_*EYE*_%) of energy contents between control and AIS groups. The energy rate of each group was obtained using energy content during quiet standing with eyes open and eyes closed

Table 1 and Fig. 5 show results comparing the energy rate between control and AIS groups for each level section. Table 1 shows quantitative results (mean ± standard deviation) of the difference in energy rate (%) between control and AIS groups. Figure 5 shows statistical results. It graphically illustrates the result of energy rate. The vertical axis shows the percentage of energy rate (*∆E*_*EYE*_%). A positive energy rate is shown when the magnitude of *E*_*EC*_% is larger than that of *E*_*EO*_% while a negative energy rate is shown in the opposite case. As a result, the energy rate of the AIS group was significantly higher than that of the control group at levels 4 to 9 (*p* < 0.05 or *p* < 0.01).

**Table 1:** Quantification result (mean ± standard deviation) of *∆E*_*EYE*_% depending on level in control and AIS groups

		Open-loop					Closed-loop					
							Somatosensory		Vestibular		Visual	
	Level	1	2	3	4	5	6	7	8	9	10	11
△E_EYE_% (%)	Control group (n = 25)	2.22 ±9.46	32.86 ±16.50	50.92 ±21.05	53.13 ±16.59	48.05 ±21.95	49.87 ±23.17	47.04 ±27.71	49.06 ±26.31	42.82 ±20.96	12.19 ±19.96	−1.91 ±9.05
	AIS group (n = 32)	− 1.49 ±11.51	26.91 ±17.47	58.47 ±21.38	63.86 ±23.48	72.78 ±25.46	72.66 ±25.56	81.33 ±24.39	79.32 ±23.39	69.55 ±21.24	−7.81 ±16.12	−17.74 ±12.34

### Analysis of sensory system between eyes open and eyes closed according to severity of AIS

Table 2 shows energy contents between eyes open and eyes closed subjects according to the severity of AIS for each level section. *E*_*EC*_% showed a significantly higher tendency than *E*_*EO*_% at levels from 4 to 9 (all *p* < 0.01). On the contrary, *E*_*EO*_% was relatively higher at level 10 (*p* < 0.05). In many frequency sections, the difference in energy contents between eyes open and eyes closed subjects was more significant in the closed-loop signal than that in the open-loop signal. Significant differences were shown in energy content (%) between eyes open and eyes closed subjects in the frequency sections of somatosensory (levels 6 and 7) and vestibular (levels 8 and 9) in all severity groups.

**Table 2:** Quantification result (mean ± standard deviation) of energy contents depending on level in AIS groups by severity

			Open-loop					Closed-loop					
								Somatosensory		Vestibular		Visual	
	Level		1	2	3	4	5	6	7	8	9	10	11
Severity (n = 32)	Mild (n = 12)	E_EO_% (%)	0.03 ±0.01	0.10 ±0.06	0.22 ±0.11	0.73 ±0.38	2.56 ±0.86	6.01 ±1.71	12.44 ±2.74	15.66 ±3.83	16.51 ±3.32	14.13 ±2.31	2.54 ±0.38
		E_EC_% (%)	0.03 ±0.01	0.12 ±0.03	0.34 ±0.11	1.23 ±0.36	4.09 ±0.72	8.81 ±1.02	18.34 ±4.99	25.23 ±6.01	25.54 ±2.67	12.85 ±1.47	2.11 ±0.76
		*p*-value	NS	NS	NS	*p* < 0.01	*p* < 0.01	*p* < 0.01	*p* < 0.01	*p* < 0.01	*p* < 0.01	*p* < 0.05	NS
	Moderate (n = 10)	E_EO_% (%)	0.04 ±0.03	0.07 ±0.03	0.15 ±0.09	0.51 ±0.33	2.03 ±0.99	7.71 ±2.91	12.52 ±3.33	14.69 ±4.58	16.92 ±3.57	13.92 ±3.07	2.02 ±0.83
		E_EC_% (%)	0.05 ±0.01	0.10 ±0.03	0.25 ±0.06	0.85 ±0.06	3.45 ±1.72	14.23 ±4.06	24.26 ±5.12	26.64 ±6.52	29.18 ±6.61	10.81 ±2.64	1.71 ±0.58
		*p*-value	NS	NS	NS	*p* < 0.01	*p* < 0.01	*p* < 0.01	*p* < 0.01	*p* < 0.01	*p* < 0.01	*p* < 0.05	NS
	Severe (n = 10)	E_EO_% (%)	0.06 ±0.05	0.10 ±0.04	0.26 ±0.02	0.61 ±0.26	2.29 ±0.65	6.57 ±1.66	9.50 ±3.15	13.69 ±3.42	15.23 ±2.66	12.88 ±1.67	2.87 ±0.99
		E_EC_% (%)	0.06 ±0.04	0.15 ±0.17	0.41 ±0.16	1.54 ±0.72	4.31 ±1.78	12.21 ±2.59	19.20 ±4.25	26.73 ±3.47	27.40 ±4.28	10.12 ±2.99	3.11 ±0.97
		*p*-value	NS	NS	NS	*p* < 0.01	*p* < 0.01	*p* < 0.01	*p* < 0.01	*p* < 0.01	*p* < 0.01	*p* < 0.05	NS

*NS* Not significant

**Fig. 6:**
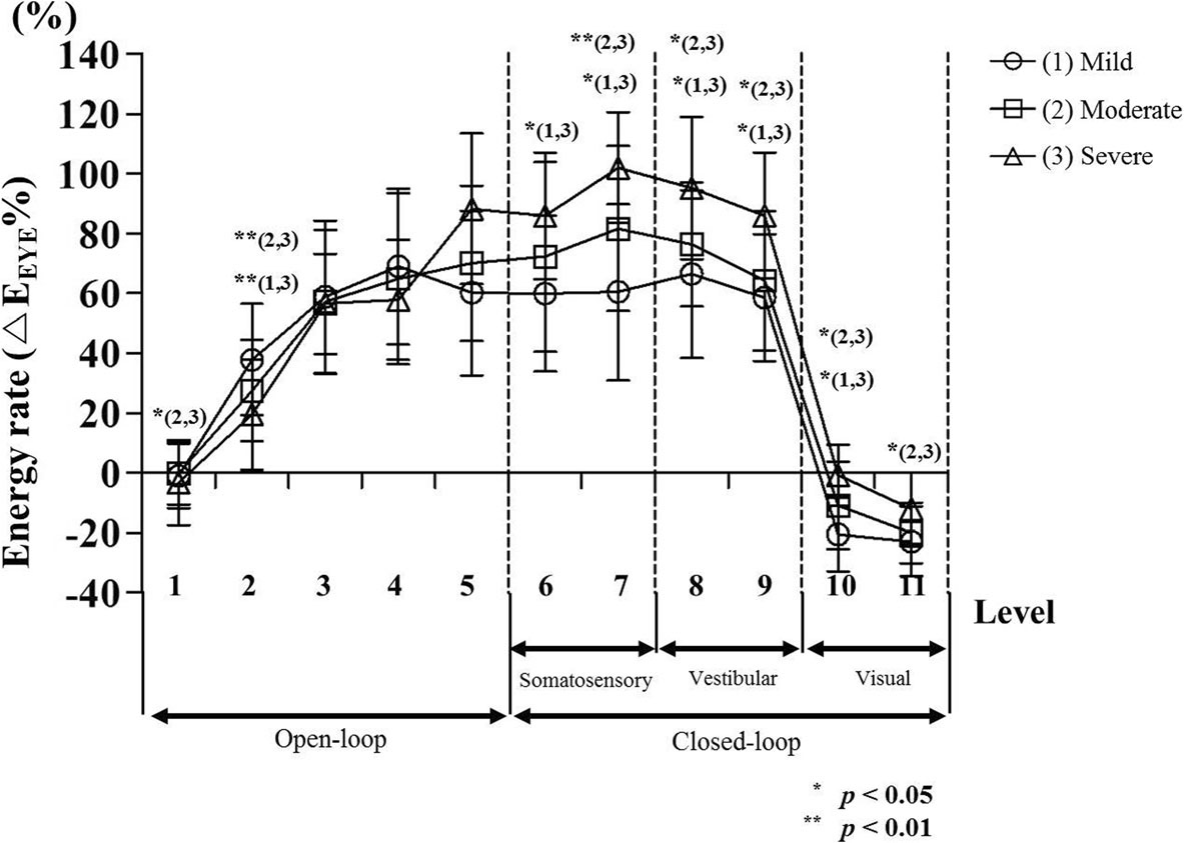
Difference in energy rate (*∆E*_*EYE*_%) of energy contents between AIS groups. The energy rate of each group classified as severity was obtained using energy content during quiet standing with eyes open and eyes closed

Table 3 and Fig. 6 show difference of energy rate (*∆E*_*EYE*_%) according to the severity of AIS for each level section. The result shows significant differences among severity groups in open-loop (level 2) and closed-loop (levels 7~ 10) signals. At levels 7 to 10, the energy rate was increased significantly depending on the severity (*p* < 0.05 or *p* < 0.01). At level 6, there was a significant difference in energy rate between mild and severe groups (*p* < 0.05). In addition, the energy rate of the severe group was increased more significantly than the moderate group at levels 1 and 11 (*p* < 0.05).

**Table 3:** Quantification result (mean ± standard deviation) of *∆E*_*EYE*_% depending on level in AIS groups by severity

		Open-loop					Closed-loop					
							Somatosensory		Vestibular		Visual	
	Level	1	2	3	4	5	6	7	8	9	10	11
△E_EYE_% (%)	Mild	−1.11 ± 10.79	37.81 ± 18.59	58.77 ± 25.55	68.95 ± 26.10	60.08 ± 27.53	59.89 ± 26.02	60.36 ± 29.43	66.42 ± 27.98	58.54 ± 21.25	−20.59 ± 12.26	−22.93 ± 11.62
	Moderate	−0.12 ± 10.51	27.49 ± 16.98	57.15 ± 23.99	64.84 ± 28.61	70.05 ± 25.96	72.22 ± 31.69	81.59 ± 27.65	76.29 ± 20.79	64.13 ± 23.25	−10.95 ± 14.63	−20.12 ± 10.10
	Severe	−3.27 ± 14.25	19.43 ± 18.47	56.48 ± 16.72	57.79 ± 19.96	88.21 ± 25.27	85.87 ± 21.20	102.04 ± 18.51	95.24 ± 23.82	85.99 ± 21.09	−0.82 ± 10.32	−12.17 ± 12.30

## Discussion

In order to analyze postural stability with respect to three sensory systems during quiet standing in AIS patients, the DWT method was applied in this study. The DWT method for CoP signal decomposition has been applied in various fields. It has been used to analyze difference of postural stability based upon gender [45], determine effect of vestibular dysfunction on postural control [46], and evaluate difference of postural control ability after cochlear implant surgery [47]. In this study, we analyzed the difference of postural instability between AIS group (*n* = 32) and control group (*n* = 25) of the similar age, and interpreted differences in sensory information in terms of visual, vestibular, and somatosensory systems. For analysis of the three sensory systems used for postural stability, we divided 11 frequency sections of the CoP signal using the DWT method [42]. The decomposed CoP signal was divided into open-loop and closed-loop signals based on 1 Hz [44]. In Figs. 3 and 4, the variation in energy content (%) showed similar tendencies in both the control and AIS groups. The energy content (%) was higher in the closed-loop than that in the open-loop signal because the feedback mechanism through neurological information played an important role in postural control [48]. In the open-loop signal, sensory feedback information is not used because the posture is directly controlled by generated motor control in the central nervous system [48, 49]. However, using only open-loop does not provide enough information to maintain posture stability. Therefore, posture control using closed-loop via sensory feedback information is needed [48, 49]. The lowest energy content was observed at level 1, which was gradually increased up to vestibular sensory system level (levels 8 and 9), and then decreased. The highest energy content appeared in levels according to vestibular input. The next highest energy content was shown in somatosensory system levels (Figs. 3 and 4). Morphological deformity of the spine can alter the head position [11, 12] which changes the CoM of trunk segment [14]. The vestibular system has a role of controlling variation of head position at the quite standing posture [11, 15]. The altered trunk CoM is rearranged by ankle and hip joints control strategy of the somatosensory system [16]. The energy content (%) showed relatively low values at visual levels. However, visual information also plays an important role in posture control as it provides continuous information to maintain a horizontal relationship between the body and the surrounding environment [17]. Figure 3c shows statistical result of the difference in energy content (%) acquired in the control group under eyes open and eyes closed conditions. Figure 4(c) shows the result for the AIS group. For control and AIS groups, significant differences in energy contents between eyes open and eyes closed conditions were shown at levels 4~ 10 (*p* < 0.05 or *p* < 0.01). At levels 4~ 9, the energy content (%) of eyes closed condition was higher than that of eyes open condition. Both groups showed higher energy content (%) in the eyes closed condition at levels 4 and 5 which corresponded to the open-loop signal. This is because the human body uses energy content (%) in the open-loop signal to compensate for body stiffness arising from the lack of visual information in eyes closed condition. The joint velocity increment for the posture rectification in eyes closed condition might increase joint stiffness [50]. Effective stochastic activity fluctuation of the open-loop control mechanisms is closely related to stiffness variations in their musculoskeletal system [51, 52]. In addition, the high reliance on eyes closed condition at somatosensory (levels 6 and 7) and vestibular (levels 8 and 9) might be compensation for the lack of visual information [37, 53]_._ On the other hand, the lower energy content (%) in eyes closed condition rather than eyes open was found at level 10 because visual information was not provided in the eyes closed condition [37].

The difference in energy rate (*∆E*_*EYE*_%) for control and AIS groups between eyes open and eyes closed conditions was analyzed (Table 1 and Fig. 5). The purpose of this analysis was to determine the effect of morphological deformation of the spine on the sensory system. Results revealed a significant difference in energy rate (*∆E*_*EYE*_%) between control and AIS groups at levels corresponding to vestibular and somatosensory systems (AIS > control; all *p* < 0.01). This study was mainly focused on the results that corresponded to vestibular and somatosensory system because the open-loop signal (levels 1~ 5) was not required for feedback information of the sensory system and the energy rate differences were very low in visual sensory levels. The vestibular system works to control the balance of the head [54]. The somatosensory system maintains postural stability through kinematic information which is generated from muscles and joints [17]. The *∆E*_*EYE*_% of the AIS group in the vestibular levels was significantly higher than that of the control group because the AIS group showed greater energy content (%) in eyes closed condition than that in eyes open condition. The human body is composed of multiple segments that are continuously moved by joints to align the body over the base of support to maintain postural stability [55–57]. However, the morphological deformity of the spine may alter the orientation of the head in 3-dimensional planes [11, 12]. The change in head position is not only the main cause of a change in trunk CoM [58]. It also changes the ability to provide information about the vestibular system [59, 60]. The human body adopts a strategy that utilizes more vestibular information in maintaining postural stability in case of visual information paucity while in eyes closed condition [37, 61]. For this reason, the *∆E*_*EYE*_% of the AIS group was higher than that of the control group in levels corresponding to the vestibular system. The *∆E*_*EYE*_% of the AIS group was also higher than that of the control group in somatosensory levels (levels 6 and 7). The morphological deformation of the spine causes an asymmetric activity of muscles between concave and convex side of spine [62]. Musculoskeletal impairments can change the head position [11, 12], alter trunk CoM position [14], and cause postural instability [62]. The somatosensory system can rearrange the altered CoM of trunk with ankle and hip joint control strategy to maintain postural stability [8, 17]. Therefore, AIS patients relied on the somatosensory system which plays a role in controlling the equilibrium of the posture through information from joints and muscles [10, 17].

In this study, we also analyzed the effect of morphological deformation of the spine on the sensory system depending on AIS severity (Tables 2, 3, and Fig. 6). Results revealed that the *∆E*_*EYE*_% had high values as morphological deformity worsened at levels 8 and 9 which corresponded to the vestibular system (*p* < 0.05). In addition, a high energy rate (*∆E*_*EYE*_%) was observed as morphological deformity worsened at somatosensory level (level 7) and visual sensory level (level 10) (*p* < 0.05 or *p* < 0.01). At level 6 (severe vs. mild) and level 11 (severe vs. moderate), energy rate difference was only observed between two severities (*p* < 0.05). The energy rate was gradually increased according to the degree of AIS severity. However, it is difficult to propose a generalized conclusion about whether a clear difference in the sensory system can maintain postural stability depending on AIS severity. This is because the standard deviation showed a large variation according to each severity level, as shown in the graph of Fig. 6. However, the energy rate (*∆E*_*EYE*_%) tended to be higher as overall severity increased. This implies that the energy content (%) of the CoP signal through the DWT method is one of important kinematic variables to distinguish AIS severity.

## Conclusion

AIS patients showed high dependency on vestibular and somatosensory systems in maintaining their postural stability compared to the control group when CoP signal was analyzed using the DWT method to determine differences in sensory systems. These results can be interpreted as compensation due to instable posture caused by asymmetry of concave and convex curves in the spine and altered head position from morphological deformity of the spine. In the present study, posture control ability was also analyzed according to the degree of AIS severity. Although it is difficult to conclude any clear difference among severities due to high standard deviation of each severity, the tendency of the energy rate according to the degree of severity differed. Evaluating CoP signals from quiet standing posture of AIS patients, regarding sensory system using DWT method can give helpful reference to compare postural stability before and after various treatments such as spinal fusion, bracing and rehabilitation. Future study is needed to deduce more generalized and concrete results by acquiring more data and interpreting with SOT. This will allow more clear interpretation of changes in physical exercise ability caused by morphological deformation of the spine.

## Abbreviations

AIS: Adolescent idiopathic scoliosis
CoM: Center of mass
CoP: Center of pressure
DWT: Discrete wavelet transform
SOT: Sensory organization test

## References

[1] Cheung CWJ, Zheng Y, Lim CT, JCH G (2010). Development of 3-D ultrasound system for assessment of adolescent idiopathic scoliosis (AIS). WCB 2010, IFMBE Proceedings.

[2] Cheung CJ, Law S, Zheng Y. Development of 3-D ultrasound system for assessment of adolescent idiopathic scoliosis (AIS): and system validation. Conf Proc IEEE Eng Med Biol Soc. 2013:6474–7. 10.1109/EMBC.2013.6611037 24111224

[3] Herman R, Mixon J, Fisher A, Maulucci R, Stuyck J (1985). Idiopathic scoliosis and the central nervous system: a motor control problem. The Harrington lecture, 1983. Scoliosis Research Society. Spine.

[4] Dobosiewicz K (1997). Neurophysiological mechanism of the unloading reflex as a prognostic factor in the early stages of idiopathic scoliosis. Eur Spine J.

[5] Nault M, Allard P, Hinse S, Le Blanc R, Caron O, Labelle H (2002). Relations between standing stability and body posture parameters in adolescent idiopathic scoliosis. Spine.

[6] Assaiante C, Mallau S, Jouve JL, Bollini G, Vaugoyeau M (2012). Do adolescent idiopathic scoliosis (AIS) neglect proprioceptive information in sensory integration of postural control?. PLoS One.

[7] Veldhuizen A, Weber D, Webb P (2000). The aetiology of idiopathic scoliosis: biomechanical and neuromuscular factors. Eur Spine J.

[8] Gur G, Dilek B, Ayhan C, Simsek E, Aras O, Aksoy S (2015). Effect of a spinal brace on postural control in different sensory conditions in adolescent idiopathic scoliosis: a preliminary analysis. Gait Posture..

[9] Haumont T, Gauchard GC, Lascombes P, Perrin PP (2011). Postural instability in early-stage idiopathic scoliosis in adolescent girls. Spine.

[10] Guo X, Chau WW, Hui-Chan CW, Cheung CS, Tsang WW, Cheng JC (2006). Balance control in adolescents with idiopathic scoliosis and disturbed somatosensory function. Spine.

[11] Chansirinukor W, Wilson D, Grimmer K, Dansie B (2001). Effects of backpacks on students: measurement of cervical and shoulder posture. Aust J Physiother.

[12] Chow DH, Kwok ML, Cheng JC, Lao ML, Holmes AD, Au-Yang A (2006). The effect of backpack weight on the standing posture and balance of schoolgirls with adolescent idiopathic scoliosis and normal controls. Gait Posture.

[13] Cheung J, Halbertsma JP, Veldhuizen AG, Sluiter WJ, Maurits NM, Cool JC (2005). A preliminary study on electromyographic analysis of the paraspinal musculature in idiopathic scoliosis. Eur Spine J.

[14] Maurer C, Mergner T, Bolha B, Hlavacka F (2000). Vestibular, visual, and somatosensory contributions to human control of upright stance. Neurosci Lett.

[15] Angelaki DE, Cullen KE (2008). Vestibular system: the many facets of a multimodal sense. Annu Rev Neurosci.

[16] Horak FB, Shupert CL, Dietz V, Horstmann G (1994). Vestibular and somatosensory contributions to responses to head and body displacements in stance. Exp Brain Res.

[17] Page P, Frank C, Lardner R. Assessment and treatment of muscle imbalance: the Janda approach. Chicago: Human Kinetics; 2010:19–25.

[18] De Santiago HA, Reis JG, Gomes MM, Da Silva Herrero CF, Defino HL, De Abreu DC (2013). The influence of vision and support base on balance during quiet standing in patients with adolescent idiopathic scoliosis before and after posterior spinal fusion. Spine J.

[19] Peterka RJ, Loughlin PJ (2004). Dynamic regulation of sensorimotor integration in human postural control. J Neurophysiol.

[20] Colnat-Coulbois S, Gauchard GC, Maillard L, Barroche G, Vespignani H, Auque J (2011). Management of postural sensory conflict and dynamic balance control in late-stage Parkinson's disease. Neuroscience.

[21] Micozzi MS. Fundamentals of complementary and alternative medicine. St. Louis: Saunders Elsevier; 2014:240–6.

[22] Mitchell DE, Dai C, Rahman MA, Ahn JH, Della Santina CC, Cullen KE (2013). Head movements evoked in alert rhesus monkey by vestibular prosthesis stimulation: implications for postural and gaze stabilization. PLoS One.

[23] Horak FB (2006). Postural orientation and equilibrium: what do we need to know about neural control of balance to prevent falls?. Age Ageing.

[24] Horak FB (2009). Postural compensation for vestibular loss. Ann N Y Acad Sci.

[25] Riemann BL, Lephart SM (2002). The sensorimotor system, part II: the role of proprioception in motor control and functional joint stability. J Athl Train.

[26] Riemann B, Lephart S (2002). The sensorimotor system, part I: the physiologic basis of functional joint stability. J Athl Train.

[27] Blackburn T, Guskiewicz KM, Petschauer MA, Prentice WE (2000). Balance and joint stability: the relative contributions of proprioception and muscular strength. J Sport Rehabil.

[28] Ting LH, McKay JL (2007). Neuromechanics of muscle synergies for posture and movement. Curr Opin Neurobiol.

[29] Beaulieu M, Toulotte C, Gatto L, Rivard C, Teasdale N, Simoneau M (2009). Postural imbalance in non-treated adolescent idiopathic scoliosis at different periods of progression. Eur Spine J.

[30] De Abreu DCC, Gomes MM, De Santiago HAR, Da Silva Herrero CFP, Porto MA, Defino HLA (2012). What is the influence of surgical treatment of adolescent idiopathic scoliosis on postural control?. Gait Posture..

[31] Oppenheim U, Kohen-Raz R, Alex D, Kohen-Raz A, Azarya M (1999). Postural characteristics of diabetic neuropathy. Diabetes Care.

[32] Kirchner M, Schubert P, Schmidtbleicher D, Haas CT (2012). Evaluation of the temporal structure of postural sway fluctuations based on a comprehensive set of analysis tools. Physica A Stat Mech Appl.

[33] Ongun N, Atalay NS, Degirmenci E, Sahin F, Bir LS (2016). Tetra-ataxiometric posturography in patients with migrainous vertigo. Pain Physician.

[34] Karapolat H, Celebisoy N, Kirazli Y, Bilgen C, Eyigor S, Gode S (2010). Does betahistine treatment have additional benefits to vestibular rehabilitation?. Eur Arch Otorhinolaryngol.

[35] Bizid R, Jully JL, Gonzalez G, François Y, Dupui P, Paillard T (2009). Effects of fatigue induced by neuromuscular electrical stimulation on postural control. J Sci Med Sport.

[36] Nagy E, Toth K, Janositz G, Kovacs G, Feher-Kiss A, Angyan L (2004). Postural control in athletes participating in an ironman triathlon. Eur J Appl Physiol.

[37] Chagdes JR, Rietdyk S, Haddad JM, Zelaznik HN, Raman A, Rhea CK (2009). Multiple timescales in postural dynamics associated with vision and a secondary task are revealed by wavelet analysis. Exp Brain Res.

[38] Sperandio EF, Alexandre AS, Liu CY, Poletto PR, Gotfryd AO, Vidotto MC (2014). Functional aerobic exercise capacity limitation in adolescent idiopathic scoliosis. Spine J.

[39] Wang WJ, Hung VWY, Lam TP, Ng BKW, Qin L, Lee KM (2010). The association of disproportionate skeletal growth and abnormal radius dimension ratio with curve severity in adolescent idiopathic scoliosis. Eur. Spine J.

[40] Lemay JF, Gagnon DH, Nadeau S, Grangeon M, Gauthier C, Duclos C (2014). Center-of-pressure total trajectory length is a complementary measure to maximum excursion to better differentiate multidirectional standing limits of stability between individuals with incomplete spinal cord injury and able-bodied individuals. J Neuroeng Rehabil.

[41] Guerraz M, Bronstein AM (2008). Ocular versus extraocular control of posture and equilibrium. Neurophysiol Clin.

[42] Kohen-Raz R, Volkman FR, Cohen DJ (1992). Postural control in children with autism. J Autism Dev Disord.

[43] Kim J, Kim C, Lee J, Kwon Y, Eom G, Tak G (2009). Human postural control against external force perturbation applied to the high-back. Int J Precis Eng Manuf.

[44] Collins JJ, De Luca CJ (1993). Open-loop and closed-loop control of posture: a random-walk analysis of center-of-pressure trajectories. Exp Brain Res.

[45] Maatar D, Fournier R, Lachiri Z, Nait-Ali A (2011). Discrete wavelet and modified PCA decompositions for postural stability analysis in biometric applications. J Biomed Sci Eng.

[46] Suarez H, Sotta G, San Roman C, Arocena S, Ferreira E, Geisinger D (2013). Postural response characterization in elderly patients with bilateral vestibular hypofunction. Acta Otolaryngol.

[47] Suarez H, Ferreira E, Alonso R, Arocena S, San Roman C, Herrera T (2016). Postural responses applied in a control model in cochlear implant users with pre-lingual hearing loss. Acta Otolaryngol.

[48] Human motor behavior: an introduction. New Jersey: Lawrence Erlbaum Associates; 2014. p. 93–116.

[49] Martin ST, Kessler M. Neurologic interventions for physical therapy. St. Louis: Saunders Elsevier; 2015:33–55.

[50] Albertsen IM, Ghédira M, Gracies JM, Hutin É (2017). Postural stability in young healthy subjects–impact of reduced base of support, visual deprivation, dual tasking. J Electromyogr Kinesiol..

[51] Lacour M, Bernard-Demanze L, Dumitrescu M (2008). Posture control, aging, and attention resources: models and posture-analysis methods. Neurophysiol Clin.

[52] Collins JJ, De Luca CJ (1995). The effects of visual input on open-loop and closed-loop postural control mechanisms. Exp Brain Res.

[53] Ray CT, Horvat M, Croce R, Mason RC, Wolf SL (2008). The impact of vision loss on postural stability and balance strategies in individuals with profound vision loss. Gait Posture..

[54] Treleaven J (2008). Sensorimotor disturbances in neck disorders affecting postural stability, head and eye movement control. Man Ther.

[55] Masso PD, Gorton GE (2000). Quantifying changes in standing body segment alignment following spinal instrumentation and fusion in idiopathic scoliosis using an optoelectronic measurement system. Spine.

[56] Hsu WL, Scholz JP, Schöner G, Jeka JJ, Kiemel T (2007). Control and estimation of posture during quiet stance depends on multijoint coordination. J Neurophysiol.

[57] Wu J, McKay S, Angulo-Barroso R (2009). Center of mass control and multi-segment coordination in children during quiet stance. Exp Brain Res.

[58] Lamontagne A, Paquet N, Fung J (2003). Postural adjustments to voluntary head motions during standing are modified following stroke. Clin Biomech.

[59] Demer JL, Oas JG, Baloh RW (1993). Visual-vestibular interaction in humans during active and passive, vertical head movement. J Vestib Res.

[60] Demer JL, Viirre ES (1996). Visual-vestibular interaction during standing, walking, and running. J Vestib Res.

[61] Schwesig R, Goldich Y, Hahn A, Müller A, Kohen-Raz R, Kluttig A (2011). Postural control in subjects with visual impairment. Eur J Ophthalmol.

[62] Zabjek KF, Coillard C, Rivard CH, Prince F (2008). Estimation of the Centre of mass for the study of postural control in idiopathic scoliosis patients: a comparison of two techniques. Eur Spine J.

